# The long reads ahead: *de novo* genome assembly using the MinION

**DOI:** 10.12688/f1000research.12012.2

**Published:** 2017-12-12

**Authors:** Carlos de Lannoy, Dick de Ridder, Judith Risse

**Affiliations:** 1 Plant Sciences, Wageningen University & Research, Wageningen, 6700AP, Netherlands; 2 Faculty of Bioscience Engineering, KU Leuven, Leuven, 3001, Belgium

**Keywords:** nanopore sequencing, MinION, \textit{de novo} sequencing, review

## Abstract

Nanopore technology provides a novel approach to DNA sequencing that yields long, label-free reads of constant quality. The first commercial implementation of this approach, the MinION, has shown promise in various sequencing applications. This review gives an up-to-date overview of the MinION's utility as a *de novo* sequencing device. It is argued that the MinION may allow for portable and affordable *de novo* sequencing of even complex genomes in the near future, despite the currently error-prone nature of its reads. Through continuous updates to the MinION hardware and the development of new assembly pipelines, both sequencing accuracy and assembly quality have already risen rapidly. However, this fast pace of development has also lead to a lack of overview of the expanding landscape of analysis tools, as performance evaluations are outdated quickly. As the MinION is approaching a state of maturity, its user community would benefit from a thorough comparative benchmarking effort of de novo assembly pipelines in the near future. An earlier version of this article can be found on bioRxiv.

## Introduction

The development of novel genome sequencing methods has been a major driving force behind the rapid advancements in genomics of the last decades. Notably, the advent of second generation sequencing (SGS) provided researchers with the required throughput and costefficiency to sequence many more genomes than was previously deemed feasible. Recent years saw the dawn of what can be considered a third generation; one that allows amplification-free reading of single DNA molecules in long consecutive stretches ^1^. Currently, this new generation is dominated by two methods: nanopore sequencing and single-molecule real time (SMRT) sequencing, championed by Oxford Nanopore Technologies (ONT) and Pacific Biosciences (PacBio), respectively.

Conceptually, nanopore sequencing is easier to explain than most other sequencing methods. An electrical potential is applied across an insulating membrane in which a single small pore is inserted. A DNA strand is pulled through the pore and the sequence is inferred from the characteristic way in which the passing base combinations influence the current. In 1989, David Deamer roughly sketched this concept as it is applied today, although it took more than two decades of key innovations to bring the concept to fruition ^2^. Since the introduction of the first commercially available nanopore sequencing device, ONT’s MinION, and the start of the MinION Access program (MAP) in 2014, the field of nanopore sequencing has been advancing at a rapid pace; both new applications and improvements to existing ones are published on a regular basis.

The advantages of the MinION over other sequencing devices are numerous. Both its size, roughly that of a cellphone, and its initial investment cost, a thousand dollars for a starter kit, are a mere fraction of that of competitors. Running the MinION is also reasonably time- and cost-effective; a 48-hour sequencing run currently costs around 800 dollars ^1^ and yields up to 5 Gbases of raw sequenced data ^3^. Furthermore, the technique does not rely on any labeling techniques to recognize different bases, while Sanger, second generation and SMRT sequencing methods do require some form of labeling of nucleotides. Amplification by PCR is optional for the MinION, while this step is mandatory for Sanger and SGSmethods. Not only does omitting these steps simplify sample preparation for MinION samples, it also helps to avoid errors and biases (e.g. the CG-bias for PCR) and allows detection of modified bases ^4^. Finally, the maximum read length produced by the MinION is many times greater than that of both second-generation and Sanger sequencing and only paralleled by SMRT sequencing, which is highly advantageous in resolving repeat sequences.

The most prominent disadvantages of the MinION, with respect to its competitors, are the lower signal-to-noise ratio, stochasticity introduced by its biological components, and the resulting high error rate of basecalling. Indeed, the MinION is a product in development and the used materials (i.e. membranes, nanopores and buffers) are still being optimized. Furthermore, it is thought that significant improvements are still possible in the software pipelines that translate current signal to DNA sequence. In this review, an up-to-date overview of *de novo* nanopore sequencing and assembly is provided. First, the physical sequencing process as it takes place inside the MinION is outlined. Then, the general structure of analysis pipelines is described, along with currently available software implemented in these pipelines and their respective strengths and weaknesses. It should be noted that nanopore sequencing is a rapidly advancing field. While some work discussed in this paper is considered cutting-edge at the moment of writing, the reader is advised to keep the publication date of said work in mind.

## 1 Physical basis of DNA sequencing using nanopores

**Figure 1.**
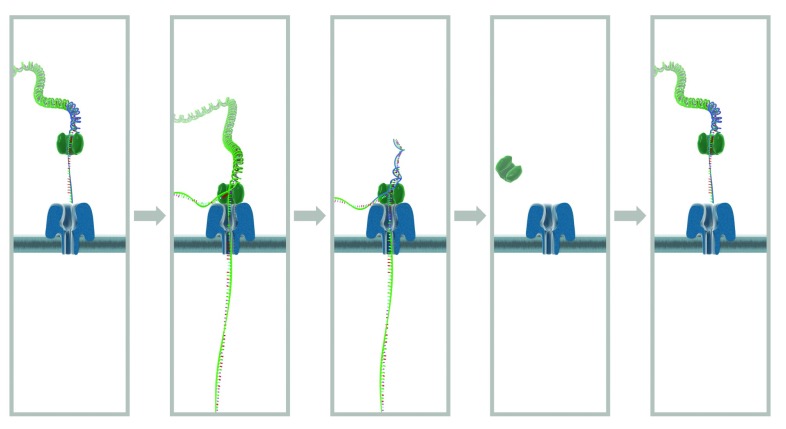
Sequencing of a DNA strand using nanopores. From left to right, double-stranded DNA with attached motor protein attaches to a pore protein in an insulating membrane. The applied potential pulls one strand through the pore, while the motor protein unzips the DNA in a step-wise fashion. After the DNA has been unzipped completely and one strand has passed through, the complex detaches from the pore entrance and the pore is ready to receive another strand. Image courtesy of Oxford Nanopore Technologies Ltd.

The underlying principle of nanopore sequencing can be explained as follows: a microscopic opening wide enough to allow single-stranded DNA to pass the nanopore is introduced in an insulating membrane between two compartments filled with saline solution and an electric potential is applied across it. DNA strands are then added to one compartment and allowed to diffuse toward the nanopore, where they are captured by the electric field and threaded through the pore. While a strand is passed through, the characteristic way in which the bases influence the electric current through the nanopore is measured. These measurements can then be decoded to retrieve the sequence of the DNA strand ( Figure 1).

In recent years, several key discoveries rapidly transformed nanopore sequencing into a usable DNA analysis method. In a step-by-step exploration of the sequencing process, these discoveries will be discussed next.

**Choice of pore: Biological versus solid-state** Nanopore sequencing efforts are sub-categorized in two groups based on the choice of nanopore. Most current efforts implement biological nanopores, which are protein multimers derived from naturally occurring counterparts. Through genetic engineering, biological nanopores are modifiable in terms of dimensions and placement of electrical charge. These properties are also highly reproducible from one pore to the next. Functionality can be further modified by attaching compatible enzymes to the pore opening. Like their naturally occurring counterparts however, they need to be embedded in a lipid membrane, which is generally prone to disruption, particularly when exposed to varying electrical potentials. In the MinION, this was partly solved by constructing membranes out of a more stable single layer of polymers, rather than the traditional bilayer. Solid-state nanopores on the other hand, are made by burning openings in a synthetic membrane using a focused electron or ion beam ^5^. Contrary to biological nanopores, solid-state nanopores are compatible with a wide range of strong and chemically stable materials with equally diverse properties. Pores are also more easily parallelized and integrated in electrical readout circuits. A major disadvantage at the moment is the irreproducibility of the pore dimensions. They also do not combine as easily with modifying enzymes. As a result, solid-state nanopores currently produce noisier and less easily interpretable signals than biological nanopores. In the following, the focus will lie on biological nanopore sequencing and the term nanopore will refer to the biological kind.

**Figure 2.**
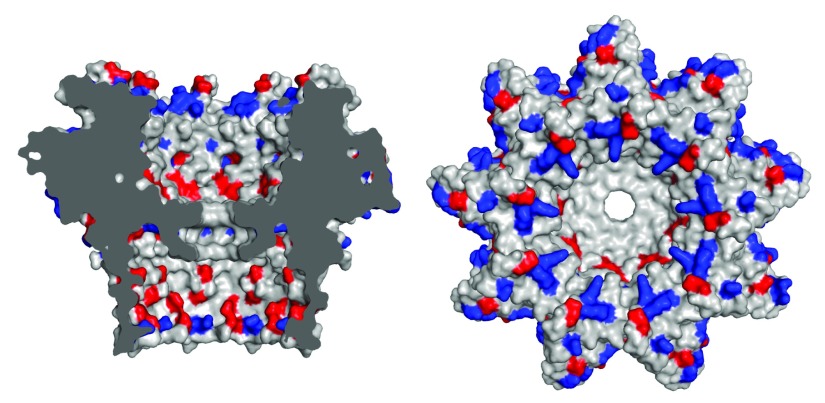
Protein structure of the CsgG pore protein complex, a variant of which is used in current generation MinION flow cells. Positive and negative residues are colored blue and red, respectively. Image generated by the authors using PyMOL v1.7.0.0. PDB ID: 4UV3 ^6^.

**Figure 3.**
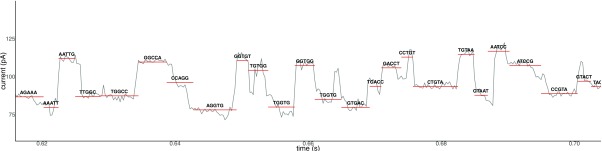
Example of a MinION DNA read as raw data (grey line) and the event data (red lines) extracted from it, corresponding to discrete sets of bases. For the sake of illustration it is assumed that five bases influence the current at a given time, although in reality this assumption may not always hold. Data used in this figure was obtained from the Nanopore WGS consortium (third release) ^3^.

**Structure and charge of the nanopore** One important structural property that makes a biological pore suitable for DNA sequencing is a constriction site at which the passing strand exerts the most influence on the electrical current. The length of the constricting passage largely determines how many bases simultaneously influence the electrical current and thus the number of bases that is “read” simultaneously at a given time. This number should be kept low enough to allow recognition of a signature current for each different combination of bases and high enough to allow for some overlap between subsequent base combinations, as this benefits basecalling accuracy by allowing every base to be read multiple times. Modified versions of both pore proteins that have seen application in the MinION, MspA (denoted by ONT with series numbers prefix “R7”) and the currently used CsgG ^6^ (denoted with prefix “R9”, Figure 2), have a constricted passage that allows detection of a manageable number of bases. For the 10Å-long constriction of the CsgG pore, basecalling models previously relied on the assumption that five nucleotides sufficiently influence the current at any given time to discern all different nucleotide combinations, and thus 5-mers were assigned to stretches of signal ( Figure 3). Although this worked reasonably well, it was found that this assumption does not always hold, e.g. due to specific base sequences and the secondary structure of the molecule influencing the current differently. Newer basecalling models therefore no longer make this assumption and assign a variable number of bases (see also section 2.1).

For sequencing to commence, a DNA strand first needs to diffuse towards one side of the pore, referred to as the cis-side, where it is captured by the electric field resulting from the applied potential. It is then threaded through the pore and extruded at the other end, called the trans-side.

Two forces should be considered. First and most importantly, the electrophoretic force induced by a positive electric potential applied at the trans-side attracts the negatively charged DNA and pulls it in. As negative particles leave the cis-side and positive particles simultaneously move in the opposite direction, a positively charged zone forms around the cis entrance of the pore, strengthening attraction of DNA strands. Secondly, strand translocation is influenced by the electro-osmotic flow (EOF), the force induced by the net water and ion flow through the pore. While a DNA strand is in the pore, the EOF normally opposes the direction of the electrophoretic force and thus of translocation; however, this effect is relatively minor.

Through iterative optimization of internal architecture, it was found that positive internal surface charges are important for efficient DNA capture ^7, 8^, while base recognition was found to improve with bulky or hydrophobic amino acid side chains placed at the constriction site, as these direct ion flow toward the DNA strand ^9^. Although the structures of the modified pores used in MinION flow cells have not been publicly released by ONT, modifications to these properties have likely been made. Currently, ONT maintains two types of flow cells containing different modified CsgG pores ^6^, designated R9.4 and R9.5. Reportedly, alterations between R9.4 and its successor R9.5 were solely made to facilitate a novel sequencing mode (dubbed 1D ^2^, see below) and should not influence sequencing accuracy in any other way. These alterations thus likely pertain to different properties of the pore.

**Processive control** It should be noted that the processive speed of the strand without any further modifications is too high for the sensor to accurately detect changes in electrical current (between 2 *·* 10 ^6^ and 10 *·* 10 ^6^ bases/s in wild-type MspA) ^7^. Currently, the most successful way to exert control over the speed has proven to be the addition of a motor protein, such as phi29 DNA polymerase ^10^ or a helicase ^11^. In a preparatory step, poly-T or “leader” adapters are attached to the doublestranded DNA. Motor proteins attach to these adapters, but due to specialized bases in the adapter sequence (possibly acridine residues as used by 12, but left unspecified by ONT ^11^), they cannot unzip it at this stage. Once one end of the complex is adjacent to the cis-side of the pore, the leader adapter previously blocking the motor protein is released, presumably due to the force exerted on the strand as demonstrated by 13 and described in 14. The DNA is then fed base-by-base through the pore by the motor protein as it processes the strand, where it can now be read at a regular pace. A modified helicase is currently used as motor protein in the MinION ^11^. The latest release of this motor protein at the time of writing (dubbed E8) maintains an average throughput speed of 450 bases/s (as noted in e.g. 3).

**Reading the DNA strand** During a MinION sequencing run, the potential over the membrane is kept stable, while the electrical current (in the pA-range) is sampled at a frequency in the kHz range ( Figure 3). This signal is characteristic for the subsequent bases moving through the pore and will ultimately serve as the basis for basecalling. As the amount of electrolyte is increasingly depleted during the run, the applied potential (typically starting at -180mV) is further decreased by 5mV per two hours of runtime and increased by 5mV when the MinION switches to another set of wells filled with fresher buffer (see next section).

**Figure 4.**
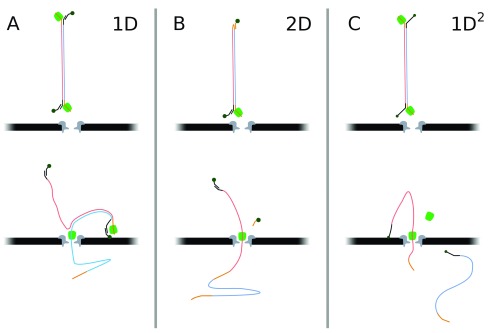
The three categories of DNA reading chemistries for the MinION. ( **A**) When using 1D chemistry, only the template strand (blue) is threaded by its motor protein (green) and read. The complement strand (red) is discarded at the cis side of the pore. The tethers (dark-green) allow for selection of properly ligated complexes during sample preparation and attach to the membrane to increase the availability of strands near pores during sequencing. ( **B**) The now-deprecated 2D chemistry connected template and complement strand using a hairpin, thus allowing sequencing of the complement strand immediately after the template strand. An additional tether that attached to the hairpin allowed for selection of correctly ligated strands during sample preparation. ( **C**) 1D ^2^ chemistry, the successor of 2D, also allows sequencing of both strands, but rather than attaching the two, the complement strand is tethered to the membrane while the template is sequenced. After the template strand is threaded through, the complement strand is drawn in and the tether is pulled loose. Based on 17 by permission from Macmillan Publishers Ltd: Nature Methods, copyright(2015), the ONT kit content description, and ONT's technical update of March 2017.

While the MinION can read the first strand of a dsDNAstretch that is threaded through the pore - by definition, the template strand - and discard the complementary strand, it is possible to instead read the complementary strand immediately after the template, thus performing a second read of the same stretch in reverse complement ( Figure 4). Combining reads of both strands has been shown to increase sequencing accuracy significantly ^15^.

The currently implemented method for doing so is referred to as 1D ^2^ sequencing (versus 1D sequencing if only the template strand is read). The 1D ^2^ chemistry provided by ONT includes different adapters that allow the complement strand to attach to the membrane while the template strand is read. Shortly after the template strand has completely left the pore, the complement strand is pulled in and sequenced. The mirrored reads are then decoded jointly so that any sequencing errors may be corrected. A previously offered method with the same aim, referred to as 2D-sequencing, involved covalently connecting the 3’-end of the template and the 5’-end of its complement using an abasic hairpin adapter, thus allowing the complement strand to be pulled in automatically after the template strand. However, due to several issues, including the hairpin’s tendency to ligate different strands into chimeric reads ^16^ and a lower read quality and sequencing speed for the complement strand ^15^ reportedly caused by secondary structure changes in the strand while rezipping after sequencing, this approach was deprecated in favor of 1D ^2^-sequencing in May of 2017.

**Figure 5.**
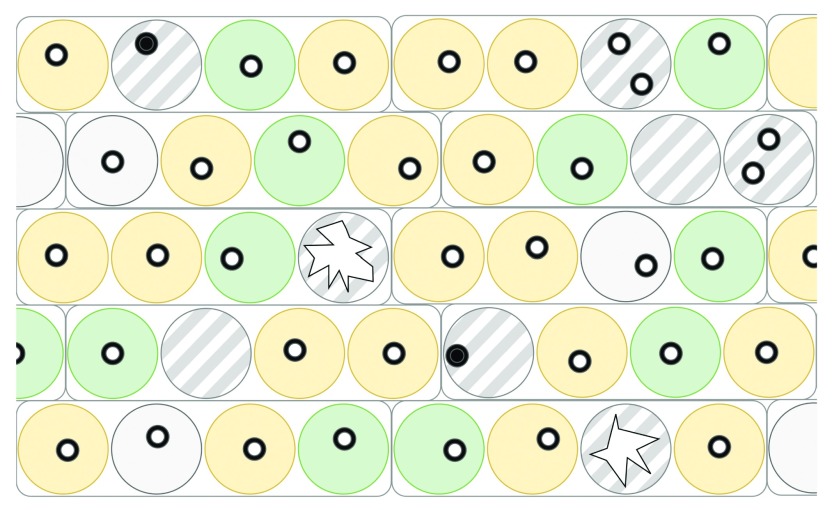
Layout of a MinION flowcell grid. Large circles denote wells in the grid, small black circles denote inserted nanopores. In reality, the pore diameter (12 nm) is much smaller with respect to the well diameter (about 10 *µ*m). Each group of four wells is controlled by a multiplexer (MUX). During an initial quality check, wells that are unusable e.g. due to erroneous pore insertion, membrane defects or pore blockades are marked as unusable (hatch pattern). Right before sequencing, the wells are tested a second time and three wells per MUX are ranked on signal quality (if possible). Sequencing of the sample will then commence, starting read-out from the best-performing well (green) and switching to second and third best (yellow) after eight hours each. The white wells are usable for sequencing, but are left unused unless the user designates otherwise in the MinION protocol.

**Channel parallelization** Lastly, throughput can be greatly increased by reading the signal from multiple pores in parallel. The current generation of the MinION’s disposable cartridges, called flow cells, can read the signal of up to 512 pores in parallel ( Figure 5). The flow cell is equipped with 2048 wells, which are connected in groups of four to multiplexers (MUXs), the switches that control which of the four cells per group is controlled and read out by the circuits. During the initial platform quality check, DNA strands (of unreleased source and sequence), present in the buffer with which the flow cells are shipped, are sequenced to discern wells suitable for sequencing (i.e. containing an intact membrane and precisely one correctly inserted, properly functioning pore) from wells in which correct pore insertion has failed ( see ONT platform quality check explanation). The latter scenario may occur, as the insertion of pores is a stochastic process. In a second quality check, the MUX scan, each MUX chooses up to three wells in order of signal quality and begins readout in the best-quality well. As well quality is expected to decline during the run, the standard protocol switches to the second-best quality pore after eight hours, and the third-best quality after another eight hours. This way, the best and most output is expected in the first part of the run. While a run using a group of wells is in progress, the circuits connected to the MUXs regulate the current in each selected well individually. This also allows expelling of eventual blockades from a pore, by temporarily reversing the current in the affected well while the rest of the wells continue to function normally.

## 2 Currently available software for MinION basecalling and *de novo* assembly

Following the process in section 1, a current signal is obtained that is subsequently translated into the underlying DNA sequence by a so-called basecaller. Next, the read sequences may be *de novo* assembled using assembly tools that can make use of the long read length while mitigating the error-prone nature of the reads. This is often followed by a last error correction or ’polishing’ step, in which a better consensus between the assembly and the raw reads is sought. In this section, these steps are detailed and a selection of available software tools to fulfill each step is explored.

### 2.1 Basecallers

Before basecalling takes place, some preparatory steps may be required. First, if the (now deprecated) 2D chemistry was used, the signal derived from the template strand should be separated from that of the hairpin and the complement strand. This process is commonly referred to as segmentation. Furthermore, older basecallers require the signal to be subdivided into discrete averaged stretches, or events, each corresponding to a particular set of *k* bases. Both segmentation and event detection can be performed by MinKNOW, the MinION control software provided by ONT. For event detection, MinKNOW was reported to calculate a simple *t*-statistic between sliding adjacent windows of set size. Peaks in the *t*-statistic above a certain threshold are then assumed to signify the borders between adjacent events.

Initially, basecallers were designed to find the most likely set of *k* bases for each event detected in this manner ^18, 19^. As it became clear that the number of bases per event is too variable for this approach, newer tools generally infer the events and the underlying sequence simultaneously from the raw signal (e.g. Albacore v *≥*2.0.1, Chiron, BasecRAWller) ^20, 21^.

To assess the quality of basecalling performance, a 3.6 kbase calibration strand derived from the Lambda genome may be added to the sample ^15, 22^. MinKNOW automatically detects reads derived from the Lambda genome and separates those from the sample reads. Software tools may also use these strands for parameter optimization (e.g. as PoreSeq does to adjust its basecall correction algorithm ^22^).

Several dedicated basecalling tools are available to MinION users. In this section, the underlying principles and implementation of these tools are explored, along with their reported strengths and weaknesses. Unfortunately, most basecallers do not support calling 1D ^2^ reads, thus performance measures will focus on 1D calling. Wick *et al.* have provided a benchmark for basecallers on a 1D, R9.4 *Kleibsiella pneumoniae* dataset generated with a SQK-LSK108 chemistry kit. To the author’s knowledge, this is currently the only comprehensive and up-to-date benchmarking effort. Comparisons made in this section are based on their analysis and reports made by the authors of the open-source basecallers in their publications. In the latter case, the used read type, pore and chemistry kit is listed between brackets each time, e.g. for the Wick *et al.* study: (1D, R9.4, SQK-LSK108).

**Metrichor basecallers** Metrichor, a spin-off company of ONT and its main developer of proprietary analysis software, maintains a range of basecallers that have remained the go-to option for most MinION users. Currently, four Metrichor basecallers are available to users: Albacore, the MinKNOW integrated basecaller, Nanonet and Scrappie. A cloud-based version was previously integrated in the EPI2ME platform, but this service has been discontinued. Both Nanonet and Scrappie are unsupported development basecallers, while Albacore and the MinKNOW version are stable tools intended for regular MinION users.

Initially, the Metrichor basecallers relied on hidden Markov models (HMMs) to assign *k*-mers of set size *k* to event-called data. As of early 2016, the HMM model was replaced by a more accurate recurrent neural network (RNN)-implementation. This approach was first introduced in Nanonet (source code publicly available), a basecaller written in Python and using the CURRENNT library ^23^ to implement its RNN. It is able to perform all steps from raw MinION signal to base sequence (i.e. segmentation, event-calling and basecalling). The next major advancement was the addition of a transducer after the RNN in April of 2017 which, rather than assigning a *k*-mer to each event, uses the newly input signal and the bases it previously emitted to determine whether to output none, one or multiple bases for the next event. Importantly, this allowed the detection of homopolymer sequences longer than a given *k*-mer size ^3^. This was previously impossible, as the sliding window *t*-test used in event detection could not discern individual events in homopolymer stretches, effectively merging them into a single event which would then be assigned a single *k*-mer ^22, 24, 25^.

From June of 2017, event-based calling was abandoned all together in favor of a more accurate raw signal-based approach. Both the transducer and raw signal-based calling were first introduced as options in Scrappie, a newer developer basecaller written in C (source code publicly available), and were later implemented in Albacore (transducer as of v1.0.1, raw signal interpretation as of v2.0.1). To date, Albacore also remains the only basecaller able to make use of 1D ^2^ reads. The MinKNOW basecaller lags slightly behind Albacore but is otherwise identical. The source code of Albacore and the MinKNOW basecaller is currently only open to developer users.

Metrichor’s up-to-date basecaller implementations (i.e. Albacore, MinKNOW and Scrappie) first center and scale the raw signal using the median signal over the entire read (as first described in 26) and then consecutively feed it through a strided convolutional filter and unidirectional RNN layers of gated recurrent units (GRUs) which receive their memory from alternating directions. The stacked unidirectional layers and use of GRUs allows the RNN to interpret the convolved signal in a long-range context from both sides, while remaining computationally efficient in use. The output of the RNN is fed into a transducer, which assigns a number of bases to each raw data point as described above ^2^. Lastly, the translocation speed of the strand is estimated using found non-homopolymeric events, which is then used to detect and correct probable collapsed homopolymer sequences.

The processing speed ^21^ and accuracy of Albacore, MinKNOW and Scrappie is currently considered to be the highest of all available basecallers. Wick *et al.* estimated median identity with the reference genome of a transducer-based raw signal-processing Albacore version (v2.0.2) at 87.6%. The introduction of raw data interpretation lead to some increase in accuracy; Albacore v2.0.1 scored 87.6% identity versus 86.5% for v1.2.6 (the last version without raw calling included by Wick *et al.*) and a similar difference was seen between Scrappie v1.1.1 processing event-called (85.8% identity) and raw data (88.1%). The effect of the introduction of the transducer at v1.0.1 can be seen in the read length, which is closer to the reference read length, and the higher corrected assembly identity, which indicates that fewer systematic errors are made. Both observations can be explained by the fact that the transducer allows for more accurate calls in homopolymer regions in particular, as was also shown by 3. As expected, the outdated Nanonet (v2.0.0) does not perform as well as Scrappie and Albacore (85.6% identity). Albacore’s median identity rate on 1D ^2^ reads has been reported by ONT at around 97%, however this has yet to be confirmed by thorough independent studies.

**Chiron** Chiron ^21^ is a third-party basecaller that shows high similarity with current Metrichor basecallers. It was written in Python and its neural network is implemented using the TensorFlow library ^27^.

Chiron first centers the raw signal around the mean and scales it over the standard deviation, after which the signal is divided up in partly overlapping batches to allow parallel processing. Much like current Metrichor basecallers, it then feeds the signal through a convolutional filter, several RNN-layers and a transducer which outputs probabilities for each base (or the absence of a base) for each raw data point. Finally, the returned base sequences for the split signal are fused into a single sequence for the entire read by finding the largest overlap.

Although Chiron’s overall structure is similar to that of Metrichor basecallers, its multiple convolutional layers, the usage of the more elaborate long short-term memory (LSTM) cells instead of GRUs and the more conventional bidirectional RNN architecture make Chiron more complex. Indeed, the benchmark published by Chiron’s authors shows that it performs slightly slower than Albacore v1.1.1 but similarly in terms of accuracy; on reads of lambda phage DNA, *E. coli* and *Mycobacterium tubercolosis* (all 1D, R9.4, SQK-LSK108), the difference between sequence identities of Albacore and Chiron did not rise above 1.2%. Albacore did do slightly better than Chiron on a human dataset generated with the same chemistry; Chiron’s authors hypothesize that this could be because Chiron was not trained on human data. These results are largely in line with the benchmark by Wick *et al.*; indeed Chiron (v0.2) performs similarly to Albacore v1.1.2, but the raw data-based Albacore v2.0.2 performs notably better. In terms of sequencing speed, Chiron’s authors showed that Albacore (2975 bases per second on a CPU) easily outperformed Chiron (21 bases per second on a CPU, 1652 on a GPU).

**BasecRAWller** While other basecallers prioritize accuracy, BasecRAWller’s ^20^ primary goal is to allow “streaming basecalling”, i.e. basecalling during sequencing directly from the raw signal. As its authors note, streaming basecalling may prove highly advantageous in selected applications, such as rejection of strands from the pore during sequencing if, based on the retrieved base sequence, it is decided that the strand is not of interest to the user. BasecRAWller is written in Python and uses the TensorFlow library ^27^ for its neural network implementation.

Like Metrichor basecallers, BasecRAWller uses a medianbased normalization method ^26^ to pre-process the raw signal. However, as the median of the signal of the entire strand (as used by Metrichor) is not available in streaming basecalling, it is approximated by using the median unoccupied pore signal, as these values were found to correlate sufficiently. The normalized signal is then consecutively fed into a unidirectional LSTM-RNN and a fully connected feed-forward network, which assigns a 4-mer to each measurement and a probability that the measurement should be recognized as the start of a new event. This information is ultimately passed on to another unidrectional LSTM-RNN which assigns zero, one or multiple bases to each event. Although bidirectional RNNs have the advantage of utilizing both past and future measurements to place a prediction in a proper context, the choice for a unidirectional network was consciously made to retain the ability to basecall in a streaming fashion.

As its authors state in their own assessment of BasecRAWller’s performance, some accuracy was surrendered to allow for streaming basecalling; Metrichor basecallers reached significantly higher accuracy on both an *E. coli* dataset (1D, R9, SQK-NSK007) and a human dataset (2D, R9.4, SQK-LSK108) (89.4% and 76% respectively, versus 82.9% and 72.5% for BasecRAWller). It should be noted that Albacore was able to take advantage of the 2D chemistry used for the human dataset, while BasecRAWller could not. Similarly, Wick *et al.* found a median identity of 74.0% for BaseCrawller (v0.1) versus Albacore’s (v2.0.2) 87.6%. An assessment by Teng *et al.* found slightly higher identity rates for BasecRAWller (v0.1) of around 82% on Lambda phage *E. coli*, *M. tuberculosis* and human datasets (all 1D, R9.4, SQK-LSK108), which were still 2% lower than that of Albacore (v1.1.1) on human data and around 8% lower for the other datasets ^21^. BasecRAWller’s authors indicate a processing speed of up to 900 bases per second using the current MinION throughput speed and sampling frequency, while Teng *et al.* indicated a maximum sequencing speed of 81 bases per second ^21^. The cause of this large difference is unclear, but important to investigate further, as a speed below 450 bases per second (the current average throughput speed of the MinION) would indicate that BasecRAWller is currently not able to function as a true streaming basecaller.

### 2.2 Assemblers

Once nanopore reads have been basecalled, they may serve several purposes. If SGS reads are available, one of several approaches to hybrid assembly (i.e. combining long error-prone and short accurate reads) may be chosen; short reads may be mapped to the nanopore reads to correct sequencing errors pre-assembly ^31^ or to create large low-error contigs. The latter goal may be achieved by using nanopore reads to close gaps and resolve repeat regions in SGS assemblies ^32^, by using them as scaffolds to properly align short reads ^33– 35^, by correcting a long read-only assembly using short reads ^36, 37^ (referred to as “polishing”, see also next section), or by creating short accurate seed regions from short reads, which are then bridged by nanopore reads ^25^. All described approaches were shown to result in accurate and highly contiguous *de novo* assemblies and in identification of repeats that were collapsed in SGS-only assemblies ^25, 31^. If no SGS reads are available, nanopore-only assembly pipelines can be used. It has been shown that using these pipelines, a cheap and highly contiguous MinION-only *de novo* draft genome can already be sequenced and assembled within one week (e.g. as was done for the 54 Mbase fungal genome of *Rhizoctonia solani* ^38^). If speed, cost or only the general structure of the genome are of major importance, a MinION-only approach may thus already be adequate. However, it should be noted that MinION-only assemblies are still generally inferior to those of hybrid methods in terms of accuracy, due to the error-prone nature of the reads ^39, 40^. If the goal is the construction of a highly accurate and contiguous assembly and SGS reads can be obtained, hybrid assemblies should be preferred. This accuracy gap is expected to diminish in the future due to the steadily increasing quality of MinION reads. With this and the cost- and time-effectiveness of the MinION in mind, the focus of this review lies on tools that can be used in *de novo* MinION-only sequencing.

As PacBio sequencers were available before nanopore sequencing had come to fruition, most assemblers able to work with MinION reads were either initially intended as PacBio tools or were written with both technologies in mind. Some tools offer specific parameter settings to account for differences in read properties between the two technologies, most importantly the differing error distributions. Giordano *et al.* showed that, on datasets of comparable size and read length distribution, assemblers consistently constructed more accurate assemblies with SMRT reads than with MinION reads (although the latter were generated with older chemistries and basecallers, see also Table 1) ^39^. While the difference in accuracy is in large part attributable to the higher number and less random distribution of sequencing errors, it does seem that those adapted for use with MinION reads are better able to mitigate its sequencing errors.

**Table 1.** Summary of comparisons between long read assemblers. ( **A**) Selected metrics for three benchmarking efforts on MinION reads, including chemistries used in the respective studies. Bold values denote the best score per metric. ( **B**) Short descriptions and reference papers for all assemblers discussed in this paper. ^1^: reads were corrected by Canu prior to assembly.

A	Judge *et al.* ^41^			Istace *et al.* ^40^			Giordano *et al.* ^39^		
	subs/ kbase	indels/ kbase	N50 (Mbase)	subs/ kbase	indels/ kbase	N50 (Mbase)	subs/ kbase	indels/ kbase	N50 (Mbase)
PBcR	1.0	12.2	1.20				0.2	17	0.616
Canu	**0.3**	**7.8**	**2.80**	**0.105**	**10.0**	0.610	**0.1**	17	0.698
SMARTdenovo				0.580	11.1	0.783	0.3	**14**	0.625
Minimap & miniasm	6.7	18.6	6.60	0.207 ^1^	13.5 ^1^	0.736 ^1^	34	67	0.739
ABruijn				0.130	10.1	**0.816**	0.1	15	**0.769**
Chemistry		MAP006			MAP005/MAP006			MAP006/007	
Read type		2D			2D			2D	
Pore		R7.3			R7.3			R7.3/R9	
Basecaller		EPI2ME			EPI2ME			EPI2ME	
Organism		*Enterobacter* *kobei*			*S. cerevisiae*			*S. cerevisiae*	
B	Description								Ref.
PBcR	Celera OLC assembler adapted for long error-prone reads.								42
Canu	The more accurate successor of PBcR.								43
SMARTdenovo	Fast and reasonably accurate assembler without prior error correction step.								Github
Minimap & miniasm	Fast assembly pipeline without error correction and consensus steps.								44
ABruijn	DBG assembler that fuses unique strings prior to assembly, produces highly contiguous assemblies.								45
TULIP	uses seed extension principle to efficiently assemble large genomes.								25
HINGE	Assesses coverage of low complexity regions prior to assembly and processes them more efficiently.								46

Assembly of MinION and SMRT reads requires a different approach than that of SGS reads; as the reads are longer, finding a correct overlap should be easier, yet they are more error-prone, which increases the uncertainty of overlaps. Because of these differences, a return of interest in overlap-layout-consensus (OLC) algorithms - which were at the peak of their popularity in the era of Sanger sequencing - is seen. Traditional De-Bruijn graph (DBG) assemblers, the more popular choice for SGS reads, were reported to return lower quality assemblies of MinION reads than OLC-based methods, but proved faster in some cases ^47^. A selection of available long read OLC and DBG assemblers is discussed in this section.

Software using traditional greedy extension algorithms (e.g. SSAKE) is rarely used in MinION read assembly as it was found to perform decidedly less well in a *de novo* assembly setting, both in terms of assembly quality and required computational resources ^47^, and is therefore not further discussed here. Furthermore, only tools that provide a full solution to their respective step in the assembly pipeline are reported here. As current assemblers either include their own error correction module ^43^ or work with uncorrected reads ^25, 44– 46^, stand-alone pre-assembly error correction tools are excluded as well. A short summary of each assembler’s characteristics and the limited number of available benchmarks is given in Table 1, although it should be noted that a proper evaluation is difficult due to the different and outdated chemistries and basecallers used. Thus, while performances noted here may provide an initial orientation in the available choice in long read assemblers, results are likely to differ when using current technology.

**PBcR & Canu** Originally developed for the first human genome draft, the Celera assembly pipeline ^48^ and its extensions ^43, 49, 50^ have remained a popular choice in a growing landscape of OLC assemblers. Briefly, the Celera assembler uses read overlaps to find contigs of which the structure can unambiguously be derived from overlap information, referred to as unitigs. It then separates unitigs that were found to occur multiple times from unique ones and attempts to orient the unique unitigs with respect to eachother. Where possible, gaps between unique unitigs are filled with non-unique unitigs. As a high read error rate is detrimental to the quality of the assembly ^51^, two different modifications to the pipeline are available. The PacBio corrected Reads (PBcR) algorithm, originally developed for the correction of PacBio reads suffering from similar error rates, uses accurate short reads mapped with high confidence to the long reads to correct errors. The assembly then proceeds as usual by Celera ^42^. Celera’s successor, Canu ^43^, provides a more accurate solution that does not require short accurate reads. Like PBcR, Canu was shown to succesfully assemble both MinION and PacBio reads ^39^. The pipeline includes three stages; correction, trimming and assembly. Overlaps are found using the efficient minhash alignment process (MHAP) ^52^, which hashes *k*-mers using different hash functions and for each hash function stores the smallest integer to which a *k*-mer of the sequence is hashed. Comparing the hashed *k*-mers per read results in initial overlap hits, which are then used to perform error correction by consensus seeking. By selecting overlaps for correction on quality, but limiting the number of overlaps a read can contribute to, Canu attempts to prevent masking of true repeat variants. Shorter reads are used at this stage to improve accuracy of longer reads. In the trimming step, overlaps are recalculated to locate and filter out regions of low coverage and high error. Reads are overlapped two more times to correct specific types of errors (i.e. missed hairpin sections for 2D reads, adapters, chimeric reads) and to adjust the error rate per overlap, before the actual assembly phase starts. With adjustments to account for erroneous alignments and residual errors, assembly essentially follows the same procedure as CABOG, another Celera-based pipeline ^49^.

Due to its thorough yet relatively efficient correction steps, Canu is significantly more accurate than both its predecessor Celera/PBcR and most other tested assemblers. In benchmarks on *Enterobacter kobei* and *S. cerevisiae* reads, it often produced an assembly with fewer indels and mismatches than others, often with higher contiguity ^39– 41^. These results are in line with the author’s own assessment ^43^.

**SMARTdenovo** SMARTdenovo is a long read OLCassembly pipeline that was originally intended to work with PacBio reads, but has been shown to produce assemblies of reasonably high continuity from MinION reads as well ^39^. Surprisingly, it does so without an error correction step prior to assembly, making SMARTdenovo a faster alternative to Canu.

As detailed on its Github page, SMARTdenovo first attempts to find read overlaps for each read in three steps at increasing accuracy by first searching hits in sorted *k*-mer tables twice and then using a banded Smith-Waterman algorithm. To find overlaps that were missed in this process, it subsequently repeats the process for pairs of reads that should overlap, given the extent to which they are overlapped by other reads. Next, low quality or chimeric read ends are identified by their decreased coverage by other reads and removed. Finally, SMARTdenovo borrows PacBio’s directed alignment graph consensus (DAGCon) algorithm ^53^ to produce the consensus assembly.

As expected, SMARTdenovo was shown to outperform Canu in terms of computing efficiency ^39, 54^. However, benchmarks on *S. cerevisiae* reads demonstrated that assemblies by Canu generally show higher identity with the reference sequence ^39, 40^. This is possibly due to the fact that the HGAP algorithm leveraged for error correction was originally intended to work with PacBio reads, which have a different error distribution. Notably, Schmidt *et al.* showed that SMARTdenovo produced an assembly of higher contiguity for the large tomato ( *Solanum pennellii*) genome and, when preceded by Canu’s pre-assembly error correction module, obtained an even more contiguous assembly with fewer predicted errors than either Canu or SMARTdenovo could, while still remaining faster than Canu alone ^54^.

**Minimap & Miniasm** In terms of speed and computational efficiency, the OLC-based pipeline consisting of Minimap and Miniasm ^44^ has a definite advantage over other existing tools ^39– 41^. This efficiency was reached through the omission of the consensus step and the use of minimizers. Much like the *k*-mer hash table used by Canu’s MHAP ^43^, a minimizer is a memory-efficient hashed representation of a sequence. Minimap computes the set of minimizers of a sequence, the “sketch”, by finding the *k*-mers represented by the smallest hash value within a certain window size of each position of the sequence. The complement of each *k*-mer is also considered. Decreasing the window size will increase the returned number of minimizers and allow for more accurate alignment, at the cost of increased computational requirements. Minimap then performs all-versus-all mapping by identifying hits between minimizers of different sequences. The found overlaps are passed on to Miniasm, which constructs an assembly graph. First, potential artefacts are removed from each read by identifying the longest stretch with a coverage of three or more other reads, and then clipping off the ends that fall outside this region. Then reads contained within other reads are removed and small bubbles, less than 50 kb in length, are popped (i.e. a consensus is taken in cases where paths split and later join up again). Finally, sequences can be extracted from stretches of the graph without multi-edges to form unitigs. The error rate at this point is practically the same as that in the raw reads, emphasizing that correct basecalling is essential for the eventual quality of the assembly. The graphical fragment assembly (GFA) output format of Miniasm conveniently allows both graphing of the uncorrected assembly and addition of consensus error correction tools, such as Nanopolish or Racon, to the pipeline.

In March of 2016, the authors of Minimap and Miniasm reported assembly of MinION reads of an *E. coli* genome in a single contig. In May of the same year, Judge *et al.* assembled an *Enterobacter kobei* genome in 16 contigs with an N50 of 662 kbase in two minutes, while the next fastest assembler (Canu) took two hours, however their benchmark showed that the omission of an error correction step caused the eventual assembly quality of *E. kobei* to be too low to properly assess by the QUAST analysis tool ^41^.

**ABruijn** While more traditional DBG assemblers performed worse than OLC assemblers on assembling long error-prone reads ^47^, the approach taken by the ABruijn assembler has shown more promise ^45^. To account for the high error rate, ABruijn filters all *k*-mers occurring in the reads by their frequency; if a *k*-mer occurs few times for given dataset and genome sizes, it is assumed that it contains basecalling errors and it is removed. Then *k*-mers are fused into so-called “solid strings”, sequences that contain no other occurring sequences as substring. The ABruijn graph is then drawn by representing solid strings as vertices and connecting them where connections exist in the reads. The edges are weighted by the number of positions between the first bases of the connected solid strings. The assembler consults the weights in this graph to quickly identify overlaps between reads, allowing to select on a minimum overlap length and maximum overhang length. The assembly graph is constructed by starting with the graph for an arbitrary read and iteratively extending it by overlapping it with other reads. ABruijn also includes an error correction routine, during which a best consensus between reads is found by identifying low-error stretches and, in between those stretches, choosing the consensus sequence that maximizes the likelihood of the read sequences.

In two independent benchmarking efforts (2D, R7.3, MAP005/006 and 2D, R7.3/R9, MAP006/007), ABruijn assembled an *S. cerevisiae* genome with higher contiguity than other included assemblers (Canu, Minimap/Miniasm, SMARTdenovo and PBcR) ^39, 40^ ( Table 1). However, ABruijn was also the only assembler to produce chimeric contigs. Furthermore, Canu’s assemblies showed higher identity with the reference genome. Thus ABruijn’s assembly routine tends to return longer contigs, while Canu is less error-prone.

**TULIP** As more reads are required to cover larger genomes, and as the time required for all-vs-all overlapping increases quadratically with an increasing number of reads, it follows that the overlap step of OLC assemblers may take unfeasibly long for very large genomes. To tackle this issue, The Uncorrected Long read Integration Process (TULIP) takes a different approach to read overlapping ^25^. Instead of all-vs-all alignment, short seed sequences are selected, which the assembler then attempts to align with long reads. This drastically cuts down the overlapping complexity and makes efficient use of long reads to cover long stretches of the genome between the seed regions. The resulting graph represents seeds as vertices and the connecting reads as edges. In a graph cleaning step, vertices with multiple in- or outgoing edges are revisited. Spurious and superfluous edges are removed aggressively, thus producing a linear graph. Note that, as the name implies, TULIP does not perform basecalling error correction.

The success of assembly using TULIP highly depends on proper seed selection. To avoid spurious connections between reads, the seeds need to be sufficiently unique in the genome and contain few sequencing errors. If available, SGS reads may be used to construct seeds, although with the increasing accuracy of MinION reads, the ends of long reads may be used as well. Apart from cutting out the need for SGS methods, the latter approach has the added advantage that pairs of seeds are connected by at least one long read. Furthermore, as TULIP is not able to assemble regions in which the gap between seeds is larger than the read length, a proper seed density over the entire genome is required. If a marker map is available for the genome, this information can be used to control the distribution of seeds in the selection process.

As a first demonstration of TULIP’s efficiency, Jansen *et al.* assembled the genome of the European eel *Anguilla anguilla* (approximately 850Mbp) with 18x coverage in three hours (excluding sequence polishing), requiring only 4.4GB of RAM and four threads ^25^. The resulting assembly was more continuous than the SGS-based reference genome. As was the case with Minimap/Miniasm however, the current quality of MinION reads combined with the lack of an error correction step necessitates post-assembly correction. The authors further showed that missed seed alignments were the most commonly encountered issue during graph simplification, followed by tangled alignments due to repetitive seeds and spurious alignments. The seeds, constructed from short SGS reads, only underwent selection by uniqueness, which did not lead to an equal distribution over the entire genome; however, density remained high enough for successful assembly. The authors noted that assembly using the tips of MinION reads as seeds proved successful for *Escherichia coli* genomes, but this has not been attempted for larger genomes yet (personal communication, May 1, 2017).

**HINGE** Although long reads provide a definite edge when attempting to resolve repeat regions, issues may still occur if not all individual repeats are spanned by at least one whole read. In such cases, HINGE may provide a solution. Rather than attempting to resolve frayed rope structures in the assembly graph afterwards, HINGE preprocesses the reads to separate repeat regions that are entirely spanned by a read (and are thus more easily resolvable) from those that are not, and collapses the latter beforehand ^46^.

First, HINGE attempts to identify reads that wholly or partly overlap a repeat region. It does so by performing all-vs-all alignment and then selecting those reads of which a stretch aligns to a proportionally larger number of other reads than the rest of the read. The intuition behind this is that reads from all copies of a repeat region existing in the genome align to each other, thus causing a characteristic abrupt increase in alignments for reads that overlap these repeat regions. Repeat regions covered entirely by at least one read can be easily resolved and are omitted from the following procedure. Of the reads lining the same repeat region, the reads that extend furthest into the repeat region (regardless of the location of the actual copy), are designated “hinges”. In the subsequent greedy extension of the hinges, the contigs will split at the hinge regions. Like Miniasm, HINGE outputs its assembly in the form of a graph. As its authors show, this is particularly useful for circular genomes.

HINGE provides an elegant solution to long repeat resolution, by separating resolvable regions from unresolvable ones beforehand. Its authors compared HINGE to Miniasm on PacBio reads of 997 circular bacterial genomes and found that overall, HINGE produced a completed genome in more cases than Miniasm could ^46^. Whether the precaution taken by HINGE is necessary is dependent on the genome under consideration and the used reads; if the genome is known to contain repeats longer than most of the reads, the described approach would be justified.

### 2.3 Post-assembly correction tools

A number of tools attempt to improve, or “polish”, assemblies by remapping long reads to the assembly and adapting the assembly to increase local resemblance to the reads. These polishing tools may be essential to use after assembly pipelines that do not include a consensus step themselves, such as Minimap/Miniasm, but have also frequently been used to polish assemblies produced by assemblers that do include this step. In this section, a selection of polishing tools is described. Notably, ONT recently published the source code for their own neural network-based polisher, Medaka. Although this tool may become a valuable addition to assembly pipelines in the future, it is currently in an early stage of development.

**Nanopolish** Nanopolish attempts to find an optimal consensus between an assembly and the raw current signal output by the MinION, by iteratively proposing and evaluating small adaptations to the assembly based on the original reads ^24^. The proposal mechanism for adaptations works in two steps. First, reads are aligned to the assembly and the resulting multiple alignment is divided in 50 bp subsequences of the assembly. For each read aligning partly or fully to a subsequence, sections in which events perfectly align to the assembly are detected. The consensus sequence between each pair of aligning sections is replaced by the aligned read subsequence, creating an initial set of alternative candidate sequences. In the second step, this set is further extended by proposing every possible one-base deletion, insertion and substitution in the previously generated candidate sequences. Of this set, the sequence maximizing the likelihood of observing the raw signal is picked. This process allows Nanopolish to explore a decent number of likely modifications, while remaining computationally tractable. As of v0.8.4, available information on methylation sites can be used to improve the quality of those sites even further. As epigenetic modifications were shown to influence the current signal ^26^, this may result in a significant improvement.

Nanopolish was found to improve assembly quality, regardless of the assembly tool used. One study on *E. coli* sequencing data reported that identity to the reference genome rose from 89% to 99% when Nanopolish (v0.4.0) was applied after Minimap/Miniasm, while improvement after Canu was more modest (98.2% to 99.6%) ^55^. Notably, the previously mentioned Wick *et al.* benchmark showed that methylation-aware polishing brought the identity of reference-based assemblies up significantly to 99.9% versus 99.7% after polishing without methylationawareness. An assessment on a *de novo* assembly has yet to be made.

Despite its efficient searching heuristic of block replacement and mutation, running Nanopolish remains a time-consuming step; in two separate benchmarking efforts, one on an *E. kobei* assembly produced by Minimap/Miniasm and one on a *S. cerevisiae* assembly by Canu, running Nanopolish (v0.4.0 and v0.5.0 respectively) required more than a month of extra CPU time ^39, 41^. Later versions of Nanopolish (especially v0.7.0 and up) were reported by its authors to work much faster.

**Racon** Racon ^56^ corrects MinION assemblies by finding a consensus sequence between reads and the assembly through the construction of partial order alignment (POA) graphs. After alignment of the reads by a mapper of choice (e.g. Minimap or Graphmap), Racon segments the sequence and finds the best alignment between a POA graph of the reads and the assembly. By default, the alignment is performed using the Needleman-Wunsch algorithm, which can align sequence and POA graph with little adaptation. The alignment process is sped up by parallelization. Racon was reported by its authors to be two orders of magnitude faster than the popular (yet currently deprecated) Nanocorrect ^24^ after assembly of an *E. coli* genome by Miniasm, albeit not quite as good at diminishing the error rate (to 1.31% versus 0.62% for Nanocorrect). Compared to consensus steps in Falcon ^57^ and Canu ^43^ on that same assembly, Racon remains an order of magnitude faster while producing similar error rates. A closer look at the remaining errors reveals that the majority consists of indels. As indel basecalling has drastically improved in newer basecallers (versus the pre-transducer basecallers used by Racon’s authors), these would likely allow Racon to reach even lower error rates. Finally, the total genome size estimate following application of Racon was closer to the reference genome size than the estimates of Canu, Falcon and Nanocorrect.

## 3 Discussion

Nanopore sequencing is a promising new venue in biology research. Inexpensive, small, capable of producing long reads and freed from the need for nucleotide labeling or amplification, it is conceivable that the MinION will make cost-effective, fast and portable *de novo* whole genome sequencing of even complex genomes possible in the future. In this review, an attempt was made to give an updated overview of the progress in this field, focusing in particular on *de novo* whole genome sequencing.

Available basecaller tools have been improving rapidly in accuracy. Notable recent improvements include the move toward raw signal-based calling and the inclusion of a transducer. For the next step in a typical sequencing routine, assembly, OLC-assemblers are currently considered the best option for accurate *de novo* nanopore-based assembly. The choice of assembler should be adapted to the characteristics of the genome and the priorities of the user. Canu is a complete and accurate solution, although SMARTdenovo was shown to be much faster against slightly diminished accuracy. The best of both methods may be obtained by combining Canu’s error correction module with SMARTdenovo. Minimap/Miniasm is by far the fastest option available, but as it lacks any form of error correction, cannot produce a usable genome draft without any post-assembly correction. For large, complex genomes, TULIP may be the more tractable alternative. Lastly, stand-alone post-assembly consensus error correction tools Nanopolish and Racon are a worthwhile addition in *de novo* sequencing pipelines and a necessity in combination with assemblers that do not contain a sequencing error correction step of their own.

Currently, the most prominent obstacle for *de novo* sequencing using the MinION is the high error rate of the reads. Improving basecalling accuracy would not only improve assembly quality in a direct manner, but may also allow more computationally efficient assembly.

The active research community surrounding the MinION has booked great progress in both the development of new applications and improvements on accuracy of existing ones. ONT also continuously works on improvements for both its hardware and software platforms, and regularly updates its users on this. Although these updates often entail welcome new features or some form of accuracy improvement, it should be noted that this policy has also lead to some difficulties. Developers may not be able to keep pace with ONT when evaluating, updating or calibrating their tools, and users may not always know which tool is suited best to their data and needs. As a result, most published studies, including tool benchmarking efforts, were conducted using older or multiple chemistries. Although such growing pains are to be expected for a novel fast-developing field of research, the MinION’s current state of development may allow for some increase in stability, thus giving the user community the time for proper evaluation.

## Notes

^1^Estimate based on a purchase of 24 flowcells and a 1 *D*/1D2 sequencing kit, 13th of October 2017

^2^A thorough discussion of neural network architectures and their respective properties is outside the scope of this article. Interested readers are referred to ^28^ and ^29^ for introductions to RNNs and convolutional networks respectively, and ^30^ for more information on transducers.
