# Supplementary material for: The long reads ahead: de novo genome assembly using the MinION
Source: F1000Res. 2017 Dec 12;6:1083. Originally published 2017 Jul 7. [Version 2] doi: 10.12688/f1000research.12012.2 (PMC5770995; doi:10.12688/f1000research.12012.2)
Supplement: Peer review history

# Peer review: Referee response for version 2

**DOI:** 10.5256/f1000research.14564.r28964

**Published:** 2018-01-16

**Authors:** Christiaan V. Henkel, Michael Liem

**Affiliations:** 1 Institute of Biology Leiden (IBL), Faculty of Science, Leiden University, Leiden, Netherlands; 2 Norwegian University of Life Sciences, Oslo, Norway

The authors have significantly improved this paper, and addressed all our previous issues and concerns. The paper is now an excellent entry point into the fast-moving and therefore sometimes confusing world of nanopore data analysis.

 We have detected no new issues that need further amendment. There are of course many further discussion points possible on the future of nanopore sequencing, but the review nicely succeeds in capturing the current state-of-the-art.

We have read this submission. We believe that we have an appropriate level of expertise to confirm that it is of an acceptable scientific standard.

# Peer review: Referee response for version 2

**DOI:** 10.5256/f1000research.14564.r28965

**Published:** 2017-12-13

**Authors:** David A. Eccles

**Affiliations:** 1 Malaghan Institute of Medical Research, Wellington, New Zealand

Carlos de Lannoy, Dick de Ridder, and Judith Risse have put in a *huge* amount of effort in improving their review of nanopore sequencing. I'm satisfied that the manuscript is now sufficiently close to describing the current state of nanopore sequencing technology, particularly when considering the level of detail present in the discussions of different aspects of the technology. While I think it's important to make reviews as current as is possible, I understand that it's also important to make sure that the information is accurate; trying to predict where ONT technology will be at the time of publication can be an exercise in futility.

 In light of this, I provide here some minor corrections / suggestions for the text. These represent my attempt at a comprehensive nit-picking of what has been discussed in the manuscript, rather than a selection of demonstrative comments as in my previous review.

**"its size, roughly that of a cellphone"**

- Nick Loman has used "office stapler" previously, which I think is a closer comparison

**"yields up to 5 Gbases of raw sequenced data"**

- The current [?unconfirmed] MinION Yield record is 18 Gb
  - https://twitter.com/ZaminIqbal/status/939234111339880448
- The current record reported in the semi-private ONT community PoreBoard is 15.7 Gb

**"second generation and SMRT sequencing methods do require some form of labeling of nucleotides"**

- Be careful with this description. The IonTorrent sequencing process doesn't use labelled nucleotides, but still depends on a specific nucleotide model due to the process of nucleotide flow during sequencing. I prefer to say that nanopore sequencing is an observational process that has a minimal dependence on models for signal capture.

**"[Amplification by PCR] is mandatory for Sanger and SGSmethods"**

- Illumina/TruSeq has an amplification-free protocol
  - https://support.illumina.com/downloads/truseq-dna-pcr-free-protocol-guide-15075699.html
- strictly, amplication is still used for that via bridge amplification on the flow cell, but that's amplifying a single short sequence per cluster, and biases associated with selective PCR amplification are therefore substantially reduced.

**"the maximum read length produced by the MinION is... only paralleled by SMRT sequencing"**

- Current read-length record for MinION is ~970kb
  - https://twitter.com/martinalexsmith/status/923894083000483840
  - even at the fastest PacBio sequencing speed of 2.5 bases/s, 970kb would take over 4 days to sequence, which is far in excess of the maximum Sequel run length. MinION sequencing time for a 970kb read is about 40 minutes (at 450 bases/s).
  - https://twitter.com/OmicsOmicsBlog/status/910163516191109120

**"Solid-state nanopores... are made by burning openings in a synthetic membrane"**

- Somewhat related: see Kerstin Göpfrich's PhD thesis, which discusses nanopores constructed from DNA, embedded into lipid membranes. It's sort of a half-way point between fully biological nanopores and solid-state nanopores.
  - https://t.co/7WtwMgM8nt

**Figure 5** is much improved; thanks.

**"much smaller with respect to the well diameter (about 10 µm)"**

- Sequencing wells are closer to 110μm in width
  - https://twitter.com/gringene_bio/status/939965835350745088
  - https://twitter.com/gringene_bio/status/938904193313554433 [from one of Clive Brown's presentations]

**"Chiron (v0.2) performs similarly to Albacore v1.1.2"**

- Note: Ryan Wick has updated his analysis to include Chiron v0.3, which produces much more accurate consensus assemblies.

**"The source code... is currently only open to developer users."**

- clarification: "users who have signed a restricted-distribution developer agreement with ONT"

**"due to the error-prone nature of the reads"**

- For now, I prefer to assume that the errors are mostly in the basecallers, rather than the reads themselves. I acknowledge that I could be wrong on this, and leave the distinction as a moot point.

**"after polishing without methylationawareness"**

 -> "after polishing without methylation-awareness"

I have read this submission. I believe that I have an appropriate level of expertise to confirm that it is of an acceptable scientific standard.

# Peer review: Referee response for version 1

**DOI:** 10.5256/f1000research.12992.r24095

**Published:** 2017-07-25

**Authors:** Christiaan V. Henkel, Michael Liem

**Affiliations:** 1 Institute of Biology Leiden (IBL), Faculty of Science, Leiden University, Leiden, Netherlands; 2 Norwegian University of Life Sciences, Oslo, Norway

This paper by Carlos de Lannoy *et al.* is a timely review of the choices available for genome sequencing based on Oxford Nanopore technology. This is a fast-moving field, and potential entrants will be well served by this exposition of the many options available. However, precisely because it is a fast-moving field, several of these options are already obsolete (yet relevant in providing context for recent developments). In addition, the many options in bioinformatics are confounded with a multiplicity of genome types (small prokaryotic to large repetitive) and nanopore chemistries.

 Many readers would benefit from additional support in navigating these multiple menageries. For this reason, we would like to ask the authors to address the following issues in more detail:

1. All methods discussed have been tested using nanopore data, but never using comparable datasets. Therefore, evaluating for example the percentages of sequence correctness is not straightforward. A table simply listing the methods with some of their key characteristics/assembly statistics/nanopore chemistries/genomes assembled would be helpful.
  Similar confusion is always looming because of the many nanopore chemistries that have been available. For example, on page 7, on the improvement of the Metrichor basecallers: the improvement here, from 68% to 95%, also coincides with the shift from R7.3 to R9. While this is mentioned as ‘numerous improvements in chemistry and hardware’, it would be clearer if the nanopore chemistry generation is explicitly mentioned whenever such percentages are listed.
2. The in-depth discussion of assembly methodology is clearly delimited to methods focusing on nanopore data only. But is this actually already a feasible option, and if not, what is needed to make this happen? All of the assembly pipelines discussed still fall short of reference genome quality. At the moment, sequence correction using short-read data (Pilon) remains necessary. Why/when should one choose for nanopore-only assembly, as opposed to hybrid?
3. Both basecalling and polishing are nanopore-specific bioinformatics tasks, however the *de novo* assemblers are in principle (and often in practice) suitable for any long-read technology. Therefore, a brief discussion comparing PacBio and nanopore for genome assembly is appropriate. PacBio-specific assemblers are briefly mentioned (SMARTdenovo, Falcon), but how do they relate to e.g. Canu?

 

 A few minor issues:

- p2, p6: please change ‘chapter’ to ‘paper’.
- p3, second column, modifications of the R9/CsgG pore: this is a good location to briefly introduce R9.4/R9.5
- p5: ‘multiple reads may be linked together by hairpins into chimeric reads’ -> multiple molecules
- Figure 5: explain that the white wells are ignored.
- p8, Canu: ‘Where possible, gaps between unique unitigs are filled with repetitive elements.’ This may need some rephrasing, ‘repetitive element’ is a specific term – not necessarily identical with ‘non-unique genome content’.
- p8: percentages for DeepNano: ‘more accurate … (approximately 70% versus 77%) and … (approximately 88% versus 87%)’. Which is which?
- Figure 7, 3rd kmer: the reverse complement of CTC isn’t TCT.
- p11: a few extra days of CPU time spent on polishing is acceptable for large genomes, but a major bottleneck for bacteria – polishing then takes longer than sequencing. Do these timings refer to prokaryotic genomes?

We have read this submission. We believe that we have an appropriate level of expertise to confirm that it is of an acceptable scientific standard, however we have significant reservations, as outlined above.

# Peer review: Referee response for version 1

**DOI:** 10.5256/f1000research.12992.r24090

**Published:** 2017-07-10

**Authors:** David A. Eccles

**Affiliations:** 1 Malaghan Institute of Medical Research, Wellington, New Zealand

Carlos de Lannoy, Dick de Ridder, and Judith Risse have written a technical review paper of MinION sequencing. This is a challenging task due to the high rate of MinION consumable obsolescence, and the breadth of use cases. Unfortunately I feel that this challenge has not been met in their paper, which appears to be a scattered mixture of discussions of differing recency with no cohesive story threading them together. I think it is especially important that a *review* paper of MinION technology is as current as possible, because it has the potential to be used extensively by others as an indication of the quality of sequencing at a particular point in time.

 I should perhaps point out that the main thing I found the most frustrating to read was a reference to R9 as current technology, and my other comments are likely coloured by me spotting this in my first scan through the article.

 Here are some additional comments about the content of the paper. This is not a comprehensive review, and should only be used as a guide to the areas of concern I have with the content:

- Title: "Coming of age" -- overlaps with Sara Goodwin's Nature Reviews Genetics paper [doi:10.1038/nrg.2016.49], which is also a review paper on sequencing technology (including nanopore). See, for example, this image:
  [https://www.nature.com/nrg/journal/v17/n6/fig_tab/nrg.2016.49_F5.html]
  A search for "coming of age" and nanopore returns another paper:
  [http://pubs.rsc.org/en/content/articlelanding/2007/mb/b702845h]
- The paper talks about "current" nanopore technology as R9, which was discontinued last year in preference to R9.4. Shortly before this, the paper discusses solid state nanopores for a review paper, when there is no commercially-available product. This is despite mentioning the introduction of 1D² technology in May 2017, which was introduced *after* R9.4 pores.
- The paper references internal nanopore community technical documentation, which is unlikely to be available to general readers of the article (and particularly not those who are considering its use).
- "The exact start and end of the hairpin is not always clear from the signal" -- I disagree with this. While current software may have difficulty interpreting the start/end, the location is obvious when looking at the raw signal. It is surprising to see this shortly before referencing our chimeric reads paper, where we give visual examples of the raw signal where the characteristic harpins are annotated.
- Figure 5: I like the idea of this figure. It's uncommon for people to give a visual indication of multiple pores and the MUX switching. However, some changes (and/or different annotation) are warranted. Using colours for both the mux type and the QC error is distracting; I would recommend using a hatch pattern to indicate failed pores. The pores are much larger than their actual size relative to the well size; this should be stated in the legend. Pores can also fail QC due to the membrane breaking, due to blocked pores, and presumably other reasons; the QC check is not only counting pores.
- Figure 6: There is enough event-annotated nanopore data available that mock data should not be necessary. Spikes and within-event signal variation that I am used to seeing in raw signal data are not present in this figure.
- "Any comparison in this chapter" -- might want to change that 'chapter' to a 'paper'.
- Canu is now published in Genome Research:
  [dx.doi.org/10.1101/gr.215087.116]
- The nanopolish algorithm has now been improved such that it takes substantially less time for v0.7:
  [http://simpsonlab.github.io/2017/06/30/nanopolish-v0.7.0/]
- The paper makes many statements around the accuracy of base-calling and assembly solutions which [mostly] ignores post-transducer base-calling. I would like to see a review paper that (if possible) concentrates on post-transducer software, which would probably mean discarding discussion on HMM-based callers.

I have read this submission. I believe that I have an appropriate level of expertise to state that I do not consider it to be of an acceptable scientific standard, for reasons outlined above.

## References

[1] GoodwinSMcPhersonJMcCombieW: Coming of age: ten years of next-generation sequencing technologies. *Nature Reviews Genetics*.2016;17(6) : 10.1038/nrg.2016.49 333-351 10.1038/nrg.2016.49 PMC1037363227184599

[2] YingL: Single molecule biology: coming of age. *Mol Biosyst*.2007;3(6) : 10.1039/b702845h 377-80 10.1039/b702845h 17533450

[3] KorenSWalenzBPBerlinKMillerJRBergmanNHPhillippyAM: Canu: scalable and accurate long-read assembly via adaptive k-mer weighting and repeat separation. *Genome Res*.2017;27(5) : 10.1101/gr.215087.116 722-736 10.1101/gr.215087.116 28298431PMC5411767

# Response

**Published:** 2017-07-12

**Authors:** Carlos de Lannoy

**Affiliations:** Faculty of Bioscience Engineering, KU Leuven, Belgium

Thank you for this thorough review. It clearly raises a number of valid points, which we will address in a revised version after other reviews have become available. We would like to point out at this time that we did intend our review to cover the most recent work, and that by “R9” we actually meant “R9.x” (R9.0-R9.5), as opposed to R7. This also explains why indeed we discuss more recent developments than R9.0, such as ID^2 sequencing. We hope this takes away some of the issues.

# Response

**Published:** 2017-07-12

**Authors:** David Eccles

**Affiliations:** Malaghan Institute of Medical Research, New Zealand

*"we did intend our review to cover the most recent work"*

Note that the author guidelines for Review articles state that "Reviews should provide a balanced and comprehensive overview of the latest discoveries in a particular field." See here. This is what I had in my mind in my decision for Approve/Reject.

It is particularly important for nanopore reviews that this is the case because ONT advances their technology and software at a very rapid rate. Academics who decide on funding for research frequently latch onto the latest review articles, as they expect those articles to be a comprehensive representation of the current state of the technology.
